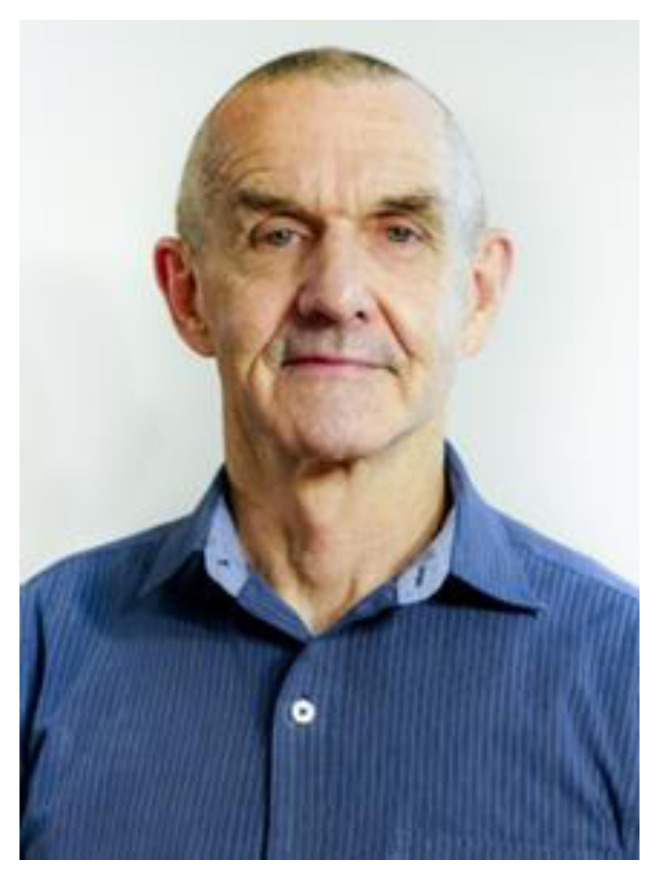# Major steps in 2021

**DOI:** 10.17159/2078-516X/2022/v34i1a13041

**Published:** 2022-01-01

**Authors:** Mike Lambert

**Affiliations:** Editor-in-chief

The South African Journal of Sports Medicine (SAJSM) took major steps in 2021. Seventy-nine submissions were received during the year with 37 submissions getting accepted for publication after review. This is a record. The 53% rejection rate may seem harsh, but not when compared to the top journal in the field (British Journal of Sports Medicine) which has a rejection rate of 78%. The SAJSM’s abstracts were viewed 8746 times, and 5064 PDFs were downloaded during the year. The highlight of the year was receiving the news that the journal passed the National Library of Medicine’s scientific quality review. The journal’s papers from 2019 onwards will be included in the NCBI’s PubMed Central database. A full listing of the other databases which cover the SAJSM is described in the index/listing section of journal.

The growth of the journal can be attributed to several factors. Firstly, the small but dedicated support staff working tirelessly to maintain the workflow from the time a paper enters the review process. Secondly, the stable platform from which to host the journal. This was provided by the Academy of Science of South Africa (ASSAf). Their management team was superb in maintaining the publication’s platform and responding to queries. This had a major impact on the growth of the journal and is aligned with their goal of ‘*growing the peer-reviewed open access scholarly journals in South Africa*’. A third factor contributing to this journal’s growth is the expanding pool of reviewers. Many reviewers were drawn from the membership of the South African Sports Medicine Association, which reflects the maturity of the organisation. Reviewers from overseas institutions were also recruited. In the majority of cases the quality of reviews was high. Reviewing is a time-consuming job for which there is no public recognition. Without these high-quality reviews it is not possible to raise the standard of the published work. It is for this reason that much gratitude is extended to all the reviewers of 2021.

Another pleasing aspect of 2021 was that the publications represented eleven institutions from around the country (Cape Peninsula University of Technology, IIE’s Varsity College, Nelson Mandela University, Stellenbosch University, Tshwane University of Technology, University of Cape Town, University of Johannesburg, University of KwaZulu-Natal, University of Pretoria, University of the Free State, University of the Witwatersrand). This shows the discipline of Sports Medicine has spread throughout the country and is not the domain of a few universities as it was a decade ago. Papers were also received from institutions in Zimbabwe, Mauritius, Kenya, England and Ireland.

There are still challenges ahead. During the COVID pandemic the “scientific process” has been stress-tested. Political influence has crept into a few high level medical journals. This has been divisive and destructive to the scientific process. Politics and science have to remain separate. Mixing the two creates a murky environment to the detriment of both. Journals have a role to play in maintaining the integrity of the scientific process. They can do this by ensuring that the review process is fair and rigorous. A rigorous review considers the research question and design of the study. Are the results believable? Do the conclusions match the results? Are any biases acknowledged? There should be no influence from outside sources with a preferred outcome of the study. These include advertisers, policy makers, and scientists invested in a particular finding. Failure of journals to uphold these basic principles of the review will contribute to the further contamination of the scientific process.

As mentioned earlier, the discipline of sport and exercise medicine has matured. But fundamental questions remain important. For example; what is the best training approach to reduce the risk of injury? What impact does injury during competition have on the athlete’s quality of life after they have retired? What are the positive effects of regular exercise on quality of life? Researchers have tried to answer these questions using measurements available at the time of the research. As new measurement techniques become available further insight into these questions are possible. The health and fitness industry has been flooded with new wearable technology with a variety of sensors (e.g. electrochemical, optical, acoustic, and pressure-sensitive). ^[1]^ Accelerometers, gyroscopes and real-time position detectors are also included in some devices. These sensors can measure heart rate, ECG, EMG, tissue oxygenation, lactate, temperature, distribution of plantar pressure, acceleration of body segments, and speed while exercising. As the sensors have become more sophisticated, the potential for greater data collection has increased. This opens opportunities for studies using machine learning and artificial intelligence. This has provided an opportunity to use big data mining analytics to answer specific questions that could not be answered in the same way previously. For example, the Fitbit company has patented an algorithm which identifies depression and predicts bipolar conditions using data (sleep, resting heart rate and voice of the user) measured with the Fitbit. ^[2]^ The accuracy of the prediction is unknown, but it indicates the potential to use a variety of measurements from wearable devices for clinical purposes.

Data scientists with expertise in artificial intelligence will be attracted to innovative applications of wearable technology using big data. There are many opportunities to revisit some of the fundamental questions and answer them using more sophisticated measurements. There is still much work to be done![Fig f1-2078-516x-34-v34i1a13041]

**Figure f1-2078-516x-34-v34i1a13041:**